# Sarcopenia: a chronic complication of type 2 diabetes mellitus

**DOI:** 10.1186/s13098-018-0326-5

**Published:** 2018-04-03

**Authors:** Heloísa Trierweiler, Gabrielle Kisielewicz, Thaísa Hoffmann Jonasson, Ricardo Rasmussen Petterle, Carolina Aguiar Moreira, Victória Zeghbi Cochenski Borba

**Affiliations:** 1 Universidade Federal do Paraná, Curitiba, PR Brazil; 2 Internal Medicine, Universidade Federal do Paraná, Curitiba, PR Brazil; 3 Statistics Department, Universidade Federal do Paraná, Curitiba, PR Brazil; 4 Endocrine Division, Hospital de Clínicas da Universidade Federal do Paraná (SEMPR), Avenida Agostinho Leão Júnior, 285, Alto da Glória, Curitiba, PR 80030-110 Brazil

**Keywords:** Pre-sarcopenia, Sarcopenia, Type 2 diabetes mellitus, Muscle weakness, DXA

## Abstract

**Background:**

Diabetics are at increased risk for impaired mobility and strength, frequently related to the disease control. Sarcopenia is the reduction of muscle mass associated with the decrease in muscle strength and/or performance, resulting in worse morbidity in chronic diseases.

**Methods:**

The objectives of this paper was to assess the prevalence of sarcopenia in patients with type 2 diabetes mellitus (T2DM) and determine its association with diabetes characteristics, progression, and complications, as well as changes in bone mineral density. The sample consisted of patients with T2DM followed at the outpatient clinics of the Serviço de Endocrinologia e Metabologia do Hospital de Clínicas da Universidade Federal do Paraná, from March to August 2016. Participants were men and women above 18 years with T2DM diagnosed at least 1 year earlier. Individuals with chronic diseases, users of any drug that modifies body composition, patients with body mass index (BMI) > 35 or < 18 kg/m^2^, and users of illicit drugs or hormonal or nutritional supplementation were excluded. The selected patients answered questionnaires about demographics, eating habits, and disease characteristics, and performed a bone densitometry exam in a dual energy absorptiometry (total body; spine and femur (total and neck)), a handgrip test by manual dynamometer, and an evaluation of the abdominal circumference (AC). The medical records were reviewed seeking diabetes data and laboratory test results. Patients were matched for sex, age, and race with healthy controls [Control Group (CG)]. The diagnosis of sarcopenia was conducted according to the criteria of the Foundation for National Institute of Health.

**Results:**

The final sample consisted of 83 patients in the DG and 83 in the CG. The DG had higher BMI, WC, past history of fractures and lower calcium and healthy diet intake (p < 0.005), compared to the CG. The DG presented a higher frequency of abnormal BMD (osteopenia in 45 (53%), and osteoporosis in 14 (19%)) and comorbidities than the CG (p < 0.005). Pre-sarcopenia was not different between groups, but muscle weakness was present in 25 diabetics (18 women) and only in 5 controls (4 men) (p = 0.00036). Sarcopenia was diagnosed in 13 (16.2%) patients in the DG and 2 (2.4%) in the CG (p = 0.01168). Pre-sarcopenia and sarcopenia were associated with altered BMD (p < 0.005), with no association with diabetes duration or control. Body mass index and osteoporosis increased the likelihood to have sarcopenia, but hypertension and healthy diet decreased it.

**Conclusion:**

The DG had altered BMD associated with worse glycemic control, and a higher prevalence of sarcopenia, suggesting the need to look for their presence in diabetics.

## Background

Diabetes mellitus is a chronic disease characterized by hyperglycemia, caused by defects in the secretion or action of insulin. According to the Brazilian Diabetes Society, there are more than 13 million diabetics in Brazil, equivalent to 6.9% of the country’s population. The global prevalence is estimated at 387 million adults, ages 20–79. More than 90% of the cases correspond to type 2 diabetes mellitus, characterized by insulin resistance with poor hormone production. Chronic hyperglycemia causes damage to the microcirculation, which impairs the functioning of various organs and tissues and predisposes to chronic complications. These complications are the result of micro- and macrovascular injuries and manifest themselves mainly as retinopathy, nephropathy, neuropathy, peripheral arterial disease, and coronary disease [1].

Damage to the skeletal muscles, with pronounced and accelerated decline in muscle quality, has been described as a new complication of diabetic patients attributed to their longer survival [2]. Sarcopenia in diabetics is associated with higher hospitalization, cardiovascular events, and mortality [3]. Sarcopenia corresponds to a progressive and generalized loss of muscular mass (pre-sarcopenia) concomitant to the decline of muscle performance, also associated with the loss of muscle strength inherent to the aging process [4]. Insulin resistance and oxidative stress are components of the pathophysiological basis of sarcopenia [5–7], which would be related to characteristic components of diabetes, such as vascular alterations [8], chronic inflammation [9], and lipid infiltration in muscles [10, 11].

From the age of 40, there is a progressive and generalized physiological loss of muscle mass, estimated at 8% per decade up to 70 years, and 15–25% per decade after this age [12]. In Brazil, the prevalence of sarcopenia among individuals over 60 years of age is 16%, corresponding to 20% of women and 12% of men [13]. Loss of muscle mass and strength is responsible for reduced mobility and increased incidence of falls and fractures, functional disability, and dependence [3, 14].

The association of diabetes and sarcopenia is described in the literature with prevalence two to three times higher in diabetics than controls [15, 16]. More important is that sarcopenia is eminently reversible, and it is possible to restore physical capacity through musculoskeletal rehabilitation [17]. Thus, the diagnosis can result in interventions that allow the prevention of lean body deterioration and better quality of life. The high prevalence among diabetic patients makes the screening for sarcopenia from middle age of great value. Improving the prevention and treatment of diabetes in the early stages would help prevent the development of sarcopenia and its complications [18, 19].

The objective of this study was to evaluate the prevalence of sarcopenia in patients with type 2 diabetes mellitus (T2DM) and its association with diabetes characteristics, progression, and complications, as well as changes in bone mineral density.

## Methods

This is a cross-sectional study approved by the Ethics Committee of the Hospital das Clínicas da Universidade Federal do Paraná under the number 53569116.7.0000.0096.

### Patients

The sample consisted of T2DM patients followed at the outpatient clinics of the Serviço de Endocrinologia e Metabologia do Hospital de Clínicas da Universidade Federal do Paraná (SEMPR), from March to August of 2016, recruited by convenience during their regular appointment. Participants (diabetes group [DG]) were men and women over 18 years of age, with T2DM diagnosed for at least 1 year, and being treated at SEMPR. Patients with other chronic disease (including type 1 diabetes, any severe or not controlled disease, any infection) users of drugs that directly modify body composition (except diabetes), users of illicit drugs, patients with body mass index (BMI) > 35 kg/m^2^ or low weight (BMI < 18 kg/m^2^), patients taking hormonal or nutritional supplementation, professional athletes and immobilized patients or mobility affected, were excluded. Patients were approached while waiting for their routine appointment at the diabetes outpatient clinic, and after verbal consent, signed the informed consent form.

Data were collected through standardized questionnaires, including demographic data (gender, age, ethnicity [white, black and Asian]); disease characteristics (time since diagnosis, presence of comorbidities, type of complications, type of drug treatment for diabetes [classified as use of one, two, or three oral antidiabetics, combined use of insulin and oral antidiabetics, or isolated insulin use]); eating habits, classified according to World Health Organization (WHO) recommendations in the Food Guide of the Brazilian Population [20], considered insufficient if the number of servings of fruits and vegetables consumed was between 0 and 6 servings per day and sufficient if more than 6 servings per day; calcium intake, estimated by accounting for the approximate amount of calcium present in milk and derivatives, classified as sufficient if daily consumption was greater than 1000 mg for patients aged 19–50 years and greater than 1200 mg for patients above 50 years. History of traumatic or non-traumatic fractures and history of smoking and alcohol intake were also investigated.

After the interview, patients were weighed on a scale (Filizola^®^), with precision of 0.1 kg, with capacity of up to 150 kg, wearing light clothes and without shoes; Waist circumference (WC) was measured, using an inextensible tape measure at the level of the umbilical scar; normal values were below 88.0 cm for women and 102.0 cm for men, as recommended by the I Brazilian Guideline for Diagnosis and Treatment of Metabolic Syndrome [21]. Stature was measured with a wall stadiometer (Tonelli^®^), with precision of 0.1 cm; the subjects were barefoot, standing on a flat surface, with their arms loose along the body, and heels were juxtaposed and leaning against the stem of the stadiometer, head upright and staring ahead [22]. The BMI was calculated and classified as recommended by the WHO [23].

All participants underwent a total body assessment for evaluation of body composition and bone mineral density (BMD) (L1–L4, femoral neck, and total femur) for analysis of bone mass using dual energy X-ray absorptiometry (DXA) on a Lunar Prodigy whole-body scanner (GE Medical Systems, PA + 302284, Madison, WI, USA). The software Encore provided data about lean body mass (bone mass plus fat-free mass), bone-free lean mass (lean mass minus fat-free mass), fat mass, and BMD.

The BMD results were expressed as g/cm^2^ and evaluated according to the recommendation of the International Society for Clinical Densitometry (ISCD) [24] and Associação Brasileira de Avaliação Óssea e Ósteometabolismo (ABRASSO) [25].

### Definition of sarcopenia

Pre-sarcopenia was defined as a low lean body mass, diagnosed by the criterion of the Foundation for the National Institute of Health (FNIH) as the appendicular lean mass (ALM) divided by the BMI (ALM/BMI); the diagnostic cutoffs are below 0.789 for men and 0.512 for women [26]. Sarcopenia was diagnosed by the concomitance of the decrease in ALM/BMI and the presence of muscle weakness, evaluated through handgrip strength using a Jamar manual dynamometer (Sammons Preston Rolyan, Bolingbrook, IL). An average of three measurements of the dominant arm was analyzed. The cut-off points for the diagnosis of muscle weakness were values lower than 26 kg for men and less than 16 kg for women.

A review of the medical records was performed searching for missing data not obtained during the interview, concerning the disease or the laboratory test results The laboratory test were performed in the routine of the clinical pathology laboratory of the Hospital de Clínicas da UFPR and the results closest to the clinical evaluation were captured from the patients files. The methods and reference values for the laboratory test were: fasting glucose (Hexoquinase/G-6-PD, NV < 100 mg/dL); calcium (Arzenazo III, NV = 8.5–10.2 mg/dL); creatinine (Jaffé, NV = 0.8–1.3 mg/day); colorimetric assays for glycated hemoglobin (NV < 6%), HDL (NV = 40–80 mg/dL) and triglycerides (NV = 50–150 mg/dL); microalbuminuria turbidimetric assay (NV < 30 mg/g) and LDL estimated by Friedwald equation (NV = 85–125 mg/dL).

### Controls

The control group (CG) comprised healthy individuals, not athletes, belonging to a database of SEMPR controls, matched for age and sex with the DG. Exclusion criteria were presence of uncontrolled chronic diseases or type 2 diabetes or glucose intolerance, or use of any medication that affects bone metabolism and/or lean mass. Individuals in this group underwent DXA and body composition at SEMPR in the years 2014 and 2015, with the same device as the DG patients and analyzed by the same professional. They answered the same questionnaires and performed the same evaluations as the patients.

### Statistical analysis

Data are presented as mean ± standard deviation (SD), unless otherwise specified. All statistical analyses were performed with R software. The normality of the distribution of the variables was evaluated with the Kolmogorov–Smirnov test. The comparison between two groups of quantitative variables was performed with Student’s *t* test for independent samples or using the nonparametric Mann–Whitney test. When comparing more than two groups, we used analysis of variance (ANOVA) with one factor and the least significant difference (LSD) test for multiple comparisons or the nonparametric Kruskal–Wallis test. For the preliminary statistical analysis, we used Fisher’s exact test and the Chi square test to assess the association between two qualitative variables. p values below 0.05 were considered statistically significant.

We performed a univariate analysis and adjusted a logistic regression model considering sarcopenia as the response (dependent) variable and BMI, dyslipidemia, HAS, healthy nutrition, osteoporosis, and past history of fractures as explanatory variables. For each variable and for the presence of the other variable included in the model, we tested the null hypothesis that the probability of sarcopenia was equal for any classification of the variable (lack of association between the variable and sarcopenia), versus the alternative hypothesis of different probabilities. The significance (p) values of the statistical tests and the odds ratio (OR) with a confidence interval of 95% were calculated.

## Results

Ninety-six patients were invited to participate in the study during their routine visit to the outpatient clinic. Of these, 90 agreed and signed the informed consent form. Seven patients were excluded because they did not meet the inclusion criteria or did meet one or more of the exclusion criteria. The final sample consisted of 83 patients (59 women), mean age of 65.84 ± 8.82 years and mean duration of diabetes of 15.55 ± 8.67 years. The CG consisted of 83 apparently healthy individuals (59 women), with mean age of 65.92 ± 8.84 years, without serious diseases. Demographic characteristics of the patients and controls are shown in Table 1.

**Table 1:** Clinical characteristics of patients and controls

	CG	DG	p value
*Age (years)*	65.92 ± 8.84	65.84 ± 8.82	0.9513
*Weight (Kg)*	66.09 ± 9.97	72.32 ± 12.00	
*Height* (m)	1.59 ± 0.08	1,6 ± 0.09	
*BMI* (Kg/m^2^)	25.96 ± 2.55*	28.16 ± 3.89*	< 0.0001
*WC* (cm)	86.20 ± 9.92	96.10 ± 11.80	< 0.0001
*Calcium intake* (mg/day)	680 ± 425*	400 ± 304*	< 0.0001
*Gender* (n (%))			1.0
Female	59 (71%)	59 (71%)	
Male	24 (29%)	24 (29%)	
*Ethnicity*			
White	81 (97.6%)	79 (95.2%)	0.4459
Black	2 (2.4%)	4 (4.8%)	
*Past history of fracture*	18 (21%)	32(38%)	0.018
*Current smoking*	1 (1.2%)	5 (6%)	0.367
*Current alcohol intake*	NA	5 (6%)	–
*Healthy diet* (portions/day)			0.01
0–3	23 (27%)	38 (45%)	
4 or more	60 (82%)	45 (54%)	

Values presented in Mean ± SD

*Kg* kilograms, *m* meters, *cm* centimeters, *n* number, *WC* waist circumference, *NA* not available

* p < 0.05

The DG presented higher BMI and WC compared to controls; 65 (78.3%) patients in the DG were overweight and had a WC above the ideal (60 [72.3%]). The majority of women evaluated were postmenopausal (58 [98%]). DG ate less healthy and had an inadequate calcium intake compared to controls.

### Past history of fractures

Previous history of fracture was observed in 32 (38.5%) patients in the DG. Atraumatic fractures in 32 (88%) of DG and in 18 (21.6%) of the CG (p = 0.018). The most frequent were ankle (25%), forearm (20%), and wrist (20%). Sixteen patients were under treatment for osteoporosis and the others (80.7%) were unaware of the diagnosis.

### Comorbidities and chronic complications

In the DG, arthrosis (26.5%), osteoporosis (21.7%), and depression (13.2%) were the comorbidities more frequently seen, and in the CG were hypertension (33%) and dyslipidemia (32%). The DG presented higher frequency of comorbidities compared to CG as, osteoporosis (19.3% vs. 7.8%), dyslipidemia (78% vs. 27%), systemic arterial hypertension (66% vs. 28%), and hypothyroidism (28% vs. 11%) (p < 0.005 for all).

Chronic complications of diabetes were present in 51 (61%) patients; the most prevalent was peripheral neuropathy (31.3%), cardiovascular disease (28.9%), and retinopathy (27.7%). The treatment varied between the use of one or more oral hypoglycemic agents combined or not, with insulin. The great majority of patients (82.1%) used one or more oral drugs (metformin and/or sulfonylurea), and 15 (17.9%) used only insulin (Table 2).

**Table 2:** Medications used and chronic complications in the DG

Chronic complications	DG absolute frequency	DG relative frequency (%)
Peripheral neuropathy	26	31.32
Cardiovascular	24	28.91
Retinopathy	23	27.71
Renal	20	24.09
Cataract	15	18.07
Medications		
*Anti-diabetic*		
1 oral hypoglycemic agent	15	18.07
2 oral hypoglycemic agents	8	9.6
Oral hypoglycemic + Insulin	45	54.21
Insulin only	15	18.07
Metformin	70	84.33
Cholesterol lowering		
Simvastatin	67	80.72

Oral hypoglycemic agent = metformin and/or sulfonylurea

*%* Percentage

### Laboratory tests

The mean laboratory results for the DG were as follows: fasting glucose 166.4 ± 68.1 mg/dL (NV < 110 mg/dL); glycated hemoglobin 8.08 ± 1.85% (NV < 6%); serum creatinine 1.16 ± 0.85 mg/dL (NV = 0.8–1.3 mg/dL); LDL 103.70 ± 39.30 mg/dL (NV = 85–125 mg/dL); HDL 45.56 ± 28.71 mg/dL (NV = 40–80 mg/day); triglycerides 147.0 ± 76.4 (NV = 50–150 mg/dL); microalbuminuria 32.3 ± 50.8 mg/g (NV < 30 mg/g); and calcium 9.56 ± 0.55 mg/dL (NV = 8.5–10.2 mg/dL). Controls had lower levels of fasting glucose 93.33 ± 19.91 mg/dL (p < 0.001); calcium 9.34 ± 0.48 mg/dL (p = 0.007); and creatinine 0.91 ± 0.2 mg/dL (p = 0.006).

### BMD results

The average BMD (g/cm^2^) in the DG was L1–L4 1.125 ± 0.214; femoral neck 0.863 ± 0.168, and total femur 0.960 ± 0.214. Altered BMD was present in 59 (71%) patients; 45 (53%) had osteopenia and 14 (19%) osteoporosis. In the CG the average BMD (g/cm^2^) was L1–L4 1.098 ± 0.23 (p = 0.197); femoral neck 0.865 ± 0.228 (p = 0.221); and total femur 0.938 ± 0.159 (p = 0.306); 48 controls (57%) had altered BMD.

In the DG the diagnosis of altered BMD was associated with past history of fractures (p = 0.053); female sex (p = 0.032); lower BMI (p < 0.001); abnormal WC (p < 0.001); and higher glycated hemoglobin (HA1C) (p < 0.001).

### Diagnosis of pre-sarcopenia and sarcopenia

The mean ALM/BMI in the DG was 0.848 ± 0.234 in males and 0.603 ± 0.225 in females, while in the CG the mean value was 0.813 ± 0.405 in males (p = 0.297) and 0.621 ± 0.382 in females (p = 0.082*).* Considering the respective cut-off values for sex, pre-sarcopenia was present in 18 (21%) diabetic patients and 21 (25%) controls (p = 0.582). Patients with pre-sarcopenia had a tendency to have an altered BMD (p = 0.054) and more frequently had lower BMI (p = 0.024), osteoporosis (p = 0.053), past history of fractures (p = 0.003), weak grip strength (p < 0.001), and an unhealthy diet (p = 0.017).

The DG presented lower muscle strength than the CG identified in 25 diabetics (18 women) and in 5 controls (4 men) (p < 0.001). The mean grip strength in the DG was 18.4 ± 5.16 and 29.39 ± 6.37 kg in women and men, respectively, and in the control group the mean value among women was 26 ± 16.45 kg (p < 0.001) and 33.92 ± 18.25 kg among men (p = 0.20).

**Fig. 1:**
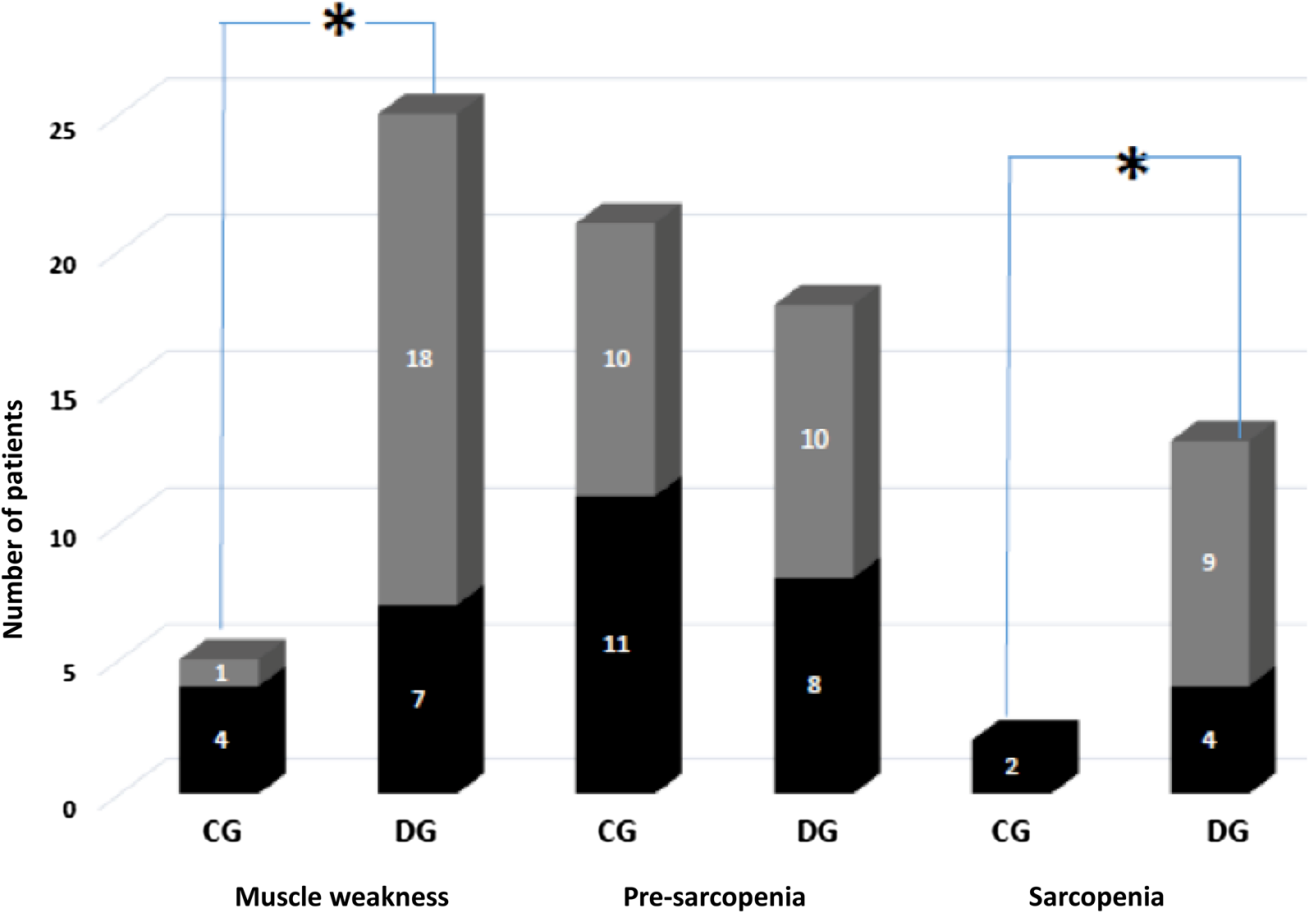
Prevalence of muscle weakness, pre-sarcopenia and sarcopenia. *p < 0.005—Fisher’s exact and Chi square tests; *CG* control group, *DG* diabetic group

Sarcopenia was identified in 13 (15.6%) diabetics and in 2 (2.4%) controls (p = 0.012) (Fig. 1).

An association was observed between sarcopenia and osteoporosis (p < 0.001), and dyslipidemia (p = 0.005); and between pre-sarcopenia and history of fractures (p = 0.029). But no association was found between glycemic controls (glycated hemoglobin), chronic complications of diabetes and sarcopenia (p > 0.05)).

**Fig. 2:**
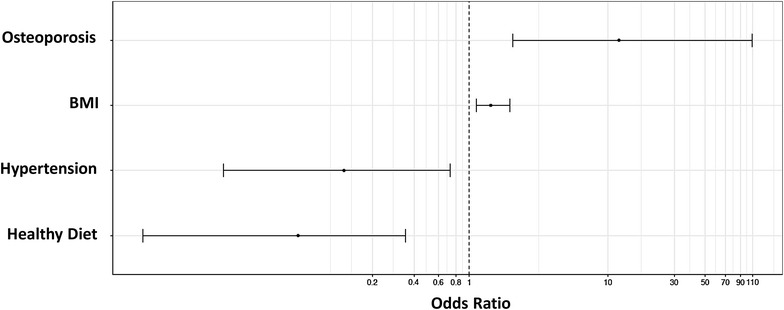
Multivariate analyses of the factors that influenced the diagnosis of sarcopenia. *BMI* Body mass index (Kg/m^2^)

The variables BMI, WC, calcium intake, dyslipidemia, hypertension, hypothyroidism, other comorbidities, unhealthy diet (< 3 healthy portions/day), osteoporosis, and past history of fractures were associated with the diagnosis of pre-sarcopenia or sarcopenia, and were tested in an univariate linear regression analysis as possible explanatory variables for the diagnosis of sarcopenia. Only dyslipidemia (p = 0.020), unhealthy diet (p = 0.016), osteoporosis (p = 0.016), and past history of fractures were significant. A multivariate analysis with a stepwise method to select the best regression equation was performed using the significant variables plus hypertension (p = 0.059), past history of fractures (p = 0.063), and BMI (p = 0.083). The results showed that BMI OR = 1.4272 (CI 25%–95% 1.280–1.9661) and the presence of osteoporosis OR = 11.9742 (CI 25%–95% 2.0514–109.0409) increased the likelihood to have sarcopenia, but the presence of hypertension OR = 1.1250 (CI 25%–95% 0.0169–0.7306) and healthy diet OR = 0.0584 (CI 25%–95% 0.0045–0.3465) decreased it (Fig. 2).

## Discussion

This study evaluated in an unprecedented way the BMD, lean mass, and muscular strength of patients with type 2 diabetes mellitus from a tertiary care outpatient clinic. A higher prevalence of muscle weakness, sarcopenia, and low bone mass was observed in these patients.

Anthropometric evaluation showed that the DG had a higher BMI and waist circumference compared to controls, with 78.3% being overweight and 72.3% with waist circumference above normal, indicating high rates of overweight and obesity. Sarcopenia and central obesity (present in the metabolic syndrome, which also exhibits insulin resistance) are frequently combined conditions in elderly individuals [27]. Together, aging and obesity are associated with progressive deterioration of muscle quality. In addition to being an important risk factor for frailty, obesity also increases the levels of inflammatory markers that inhibit the synthesis of muscle proteins [26]. The association found between pre-sarcopenia and high body mass index is also described in other studies [28, 29].

The literature describes a process named diabetic paradox, which is the normal BMD and increased risk of fractures in diabetics [28]. The genesis may involve direct or indirect effects of glycaemia on bone cells, hypovitaminosis D, hormonal factors, and obesity [29]. Although the literature does not consider DM2 a risk factor for osteoporosis or metabolic bone disease [30], DM2 patients in this study presented BMD alterations in 71%, osteoporosis in 19%, and a tendency to associate BMD with the past history of fractures. Another important finding was the association between altered BMD with worse glycemic control, measured by glycated hemoglobin levels, an index of the diabetes severity, already suggested by others [30–32].

Muscle performance evaluated by dynamometry showed a five times higher muscle weakness among diabetics, indirectly suggesting a greater impairment of muscle quality. Sarcopenia was diagnosed in 16% of the DG, eight times more prevalent than in the GC. The main mechanisms proposed to explain the greater susceptibility of diabetics to muscle mass deterioration are related to the pathogenesis of sarcopenia, such as reduction in the synthesis or sensitivity of anabolic hormones [5], cytokine secretion, chronic inflammatory state, and induced mitochondrial dysfunction by chronic hyperglycemia [19].

Patients with sarcopenia have higher rates of falls and fractures [33]. In this study, there was a relationship between pre-sarcopenia with a history of fractures and association with low BMD, suggesting that body composition and muscle function may play a synergistic role in the mechanism of falls and fractures in patients with diabetes. The association between sarcopenia and altered BMI is an aggravating factor for the morbidity of the condition. Lower muscle strength and poor ability in physical performance tests are risk factors for falls [30], and the risk of fractures is even greater in patients with compromised bone quality.

Diabetics had poorer eating habits than controls. Calcium intake was 3.5 times lower in the diabetic group. Consumption of milk and dairy products is associated with a reduction in risk of developing diabetes [34], glucose regulation, and better diabetes management [35, 36]. Frequent consumption of fruits and vegetables helps prevent the development of sarcopenia in the elderly [37, 38]. In this study, patients who ate less healthy food had more pre-sarcopenia. Oxidative stress is considered one of the main pathogenetic mechanisms of sarcopenia. A diet rich in fruits and vegetables could provide antioxidants and reduce the damage caused by the inflammatory status, reducing the risk of developing the disease [38].

Comorbidities are very common in diabetic patients. Dyslipidemia is one of the most common comorbidities related to T2DM [39, 40] and was observed in 94% of the DG. This massive prevalence increases the risk of cardiovascular disease and premature death. An association between sarcopenia and dyslipidemia was found, a result consistent with studies that verified the influence of obesity and sarcopenia on worsening dyslipidemia [40, 42]. Systemic arterial hypertension is twice as prevalent in diabetics compared to the general population and is present in 50% of patients at the time of diagnosis of T2DM [41–43]. A higher prevalence of 79.5% of hypertension was observed in the GD, more than double that observed in controls. On the other hand, the presence of hypertension in this specific population decreased the possibility of sarcopenia, consistent with the suggestion that not the disease but its treatment with beta-blockers or renin-angiotensin aldosterone inhibitors could prevent muscle wasting [44, 45].

Hypothyroidism, present in 33.7% of the GD, was much more frequent in this group than in the CG. Diabetics are more likely to have hypothyroidism when compared to the healthy population [46, 47]. Hypothyroidism also aggravates the complications of diabetes, such as peripheral arterial disease, nephropathy, retinopathy, diabetic neuropathy [46], and metabolic disorders, such as dyslipidemia, which may contribute to high mortality due to ischemic cardiovascular disease in T2DM [48].

Diagnosis of pre-sarcopenia (decrease in lean mass) was similar between the groups, suggesting that in this population, the quantitative analysis of muscle mass alone is not a good marker of muscle quality. Although muscle mass is a contributory factor for muscle strength, evidence shows that muscle performance is more related to the muscle function [49].

Deterioration of lean mass and muscle function was clearly more pronounced among diabetic patients compared to controls, although it was not possible to establish a relationship between sarcopenia and diabetes performance, evaluated by diabetes duration, glycemic control, and presence of chronic complications. Similarly, the literature has shown that the greatest decline in muscle mass and function among elderly patients with T2DM occurs independently of disease duration, metabolic control, and the presence of chronic complications [48]. However, other studies defend the relevance of this association [5, 50–52].

In the DG the diagnosis of altered BMD was associated with past history of fractures (p = 0.053); female sex (p = 0.032); lower BMI (p = 0.0076); abnormal WC (p = 0.00027); and higher glycated hemoglobin (HA1C) (p < 0.0001).

Although similar in the DG and CG, BMD in the DG was associated with traditional risk factors such as past history of fractures, female sex, and lower BMI and specific for this population with worse glycemic control and abnormal WC, which points to the need to evaluate the risk factors for fracture and perform densitometric evaluation in these patients. The impact of pre-sarcopenia evaluation still needs to be better determined; however, higher prevalence of sarcopenia suggests the importance of the evaluation of muscular function through dynamic tests such as a simple measurement of handgrip by dynamometry. The early diagnosis of both diseases would enable preventive measures to increase strength and prevent falls and fractures, which would have an impact on the quality of life and morbidity of diabetic patients.

The limitations of this study were the lack of the performance evaluation and the absence of a healthy population without abnormal glucose metabolism with similar BMI (30 and 35 kg/m^2^) to compare groups with the same body composition.

In conclusion, this population of T2DM had higher prevalence of sarcopenia than a non-diabetic population. Muscle involvement occurred in varying degrees, regardless of the characteristics of the diabetes. The recognition of individuals at risk could urge preventive measures and treatment by the primary care provider to prevent lean mass deterioration and promote better quality of life for patients with type 2 diabetes mellitus.

## Abbreviations

T2DM: type 2 diabetes mellitus
SEMPR: Serviço de Endocrinologia e Metabologia do Hospital de Clínicas da Universidade Federal do Paraná
BMI: body mass index
DXA: dual energy absorptiometry
AC: abdominal circumference
CG: control group
FNIH: Foundation for National Institute of Health
DG: diabetes group
BMD: bone mineral density
ISCD: International Society for Clinical Densitometry
ABRASSO: Associação Brasileira de Avaliação Óssea e Ósteometabolismo
FNIH: Foundation for the National Institute of Health
ALM: appendicular lean mass
SD: standard deviation
ANOVA: analysis of variance
LSD: least significant difference
OR: odds ratio
Kg: kilograms
M: meters
Cm: centimeters
N: number
WC: waist circumference
NA: not available
%: percentage
NV: normal values
HA1C: glycated hemoglobin
